# A comprehensive census of horizontal gene transfers from prokaryotes to unikonts

**DOI:** 10.1186/gb-2011-12-s1-p20

**Published:** 2011-09-19

**Authors:** Pere Puigbò, Sergei Mekhedov, Yuri I Wolf, Eugene V Koonin

**Affiliations:** 1 National Center for Biotechnology Information, National Library of Medicine, National Institutes of Health, Bethesda, MD, USA

## Background

Horizontal gene transfers (HGTs) are pervasive in prokaryotes [1], being the routes of net-like evolution that collectively dominate the evolution of prokaryotes [2]. However, in eukaryotes, the effect of HGT has not been thoroughly analyzed, with the exception of the massive HGT from the endosymbionts [3]. Here, we report a comprehensive analysis of likely HGT events in different groups of unikonts (Amoebozoa, Archamoebae, Mycetozoa, the Fungi/Metazoa group, Choanoflagellida, Fungi and Metazoa).

## Methods

We analyzed the complete proteomes of 36 species of unikonts: 1 from the Archamoebae, 1 from Mycetozoa, 18 from Fungi, 13 from Metazoa and 1 from Choanoflagellida. These proteomes were manually selected to widely represent the unikont supergroup. Initial pre-candidate genes were obtained by analyzing each proteome using the DarkHorse program [4]. The program BLASTClust was then used to make clusters of putative unique transfer events at the origin of the different groups of unikonts. These clusters were separated into two groups: group I candidate clusters (clusters with no eukaryotic representative other than the unikont group analyzed), and group II candidate clusters (clusters with representatives from prokaryotes, the unikont group analyzed and other eukaryotes). Sequences from group I candidate clusters were analyzed using BLAST versus nr and RefSeq databases, compared with the clusters of orthologous groups for eukaryotic complete genomes (KOGs) [5] and manually curated to remove false positives that result from bacterial contamination of the genomic DNA. Group II candidate clusters were analyzed using a series of automatic, conservative filters to assess the quality of the candidates. Finally, all clusters were phylogenetically analyzed to define the final candidates and to infer putative donors.

## Results

Using this methodology, we detected numerous probable HGT events from prokaryotes (mainly Bacteria) to unikonts. These events are not distributed uniformly throughout the evolution of unikonts: for example, almost all HGTs detected in Amoebozoa occurred after the divergence of Archamoebae and Mycetozoa. Importantly, we also detected many HGT events from Bacteria to Fungi, Choanoflagellida and Metazoa.

## Conclusions

Although HGTs are not as pervasive in eukaryotes as in prokaryotes, the amount of HGT detected in this study suggests that the acquisition of genes from Bacteria played a major role in the evolution of the unikonts.
